# Supplementary material for: Spatiotemporal single-cell RNA sequencing of developing chicken hearts identifies interplay between cellular differentiation and morphogenesis
Source: Nat Commun. 2021 Mar 19;12:1771. doi: 10.1038/s41467-021-21892-z (PMC7979764; doi:10.1038/s41467-021-21892-z)
Supplement: Supplementary file 1 — Supplementary Information [file 41467_2021_21892_MOESM1_ESM.pdf]

Supplemental Information for:

Spatiotemporal single-cell RNA sequencing of developing chicken hearts identifies interplay between cellular differentiation and morphogenesis

Authors: Madhav Mantri[1,2,#], Gaetano J. Scuderi[1,#], Roozbeh Abedini-Nassab[1,3], Michael F.Z. Wang[1,2], David McKellar[1], Hao Shi[1], Benjamin Grodner[1], Jonathan T. Butcher[1,*], and Iwijn De Vlaminck[1,*]

Affiliations: [1]Nancy E. and Peter C. Meinig School of Biomedical Engineering, Cornell University, Ithaca, New York, USA, [2]Computational Biology Ph.D. Program, Cornell University, Ithaca, New York, USA, [3]Department of Engineering, University of Neyshabur, Neyshabur, Iran.

*To whom correspondence should be addressed: vlaminck@cornell.edu
[#]These authors contributed equally

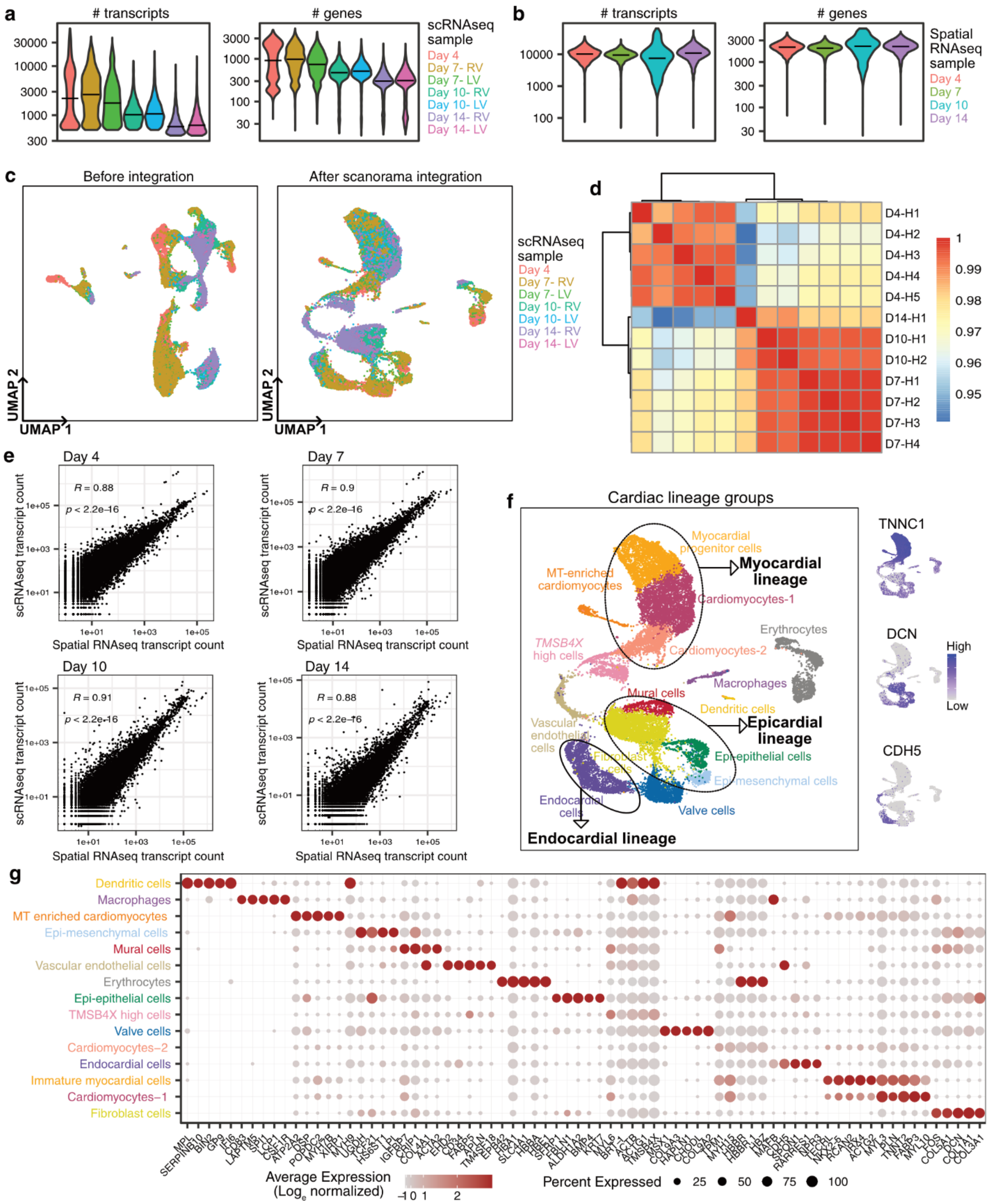

Supplementary Figure 1: Time matched scRNA-seq and spatial RNA-seq atlas of developing embryonic chicken hearts. a) Number of unique transcripts per cell (left) and number of unique genes detected per cell (right) in seven scRNA-seq samples across four developmental stages. **b)** Number of unique transcripts per spot (left) and number of unique genes detected per spot (right) in four spatial RNA-seq samples across four developmental stages. **c)** UMAP projection of 22,315 scRNA-seq cells color labeled by scRNA-seq samples before (left) and after (right) scanorama based integration and batch effect removal. **d)** Pearson correlation (two-sided) between total spot gene

expression in individual hearts (12 hearts) across 4 developmental stages in spatial RNA-seq data. Gene expression in hearts within the same developmental stage correlated well with each other (Pearson; R > 0.97). **e)** Pearson correlation (two-sided) between total gene expression in scRNA-seq samples and spatial RNA-seq samples from the same development stage (left to right: day 4 to day 14 stages). **f)** UMAP projection of 22,315 scRNA-seq cells clustered by gene expression and color labelled by cell types with black circles highlighting the endocardial, epicardial, myocardial lineages (left). Feature plots showing expression of lineage specific markers: *TNNC1* for myocardial, *DCN* for epicardial, and *CDH5* for endocardial (right). **g)** Top 5 differentially expressed genes for all clusters in scRNAseq data.

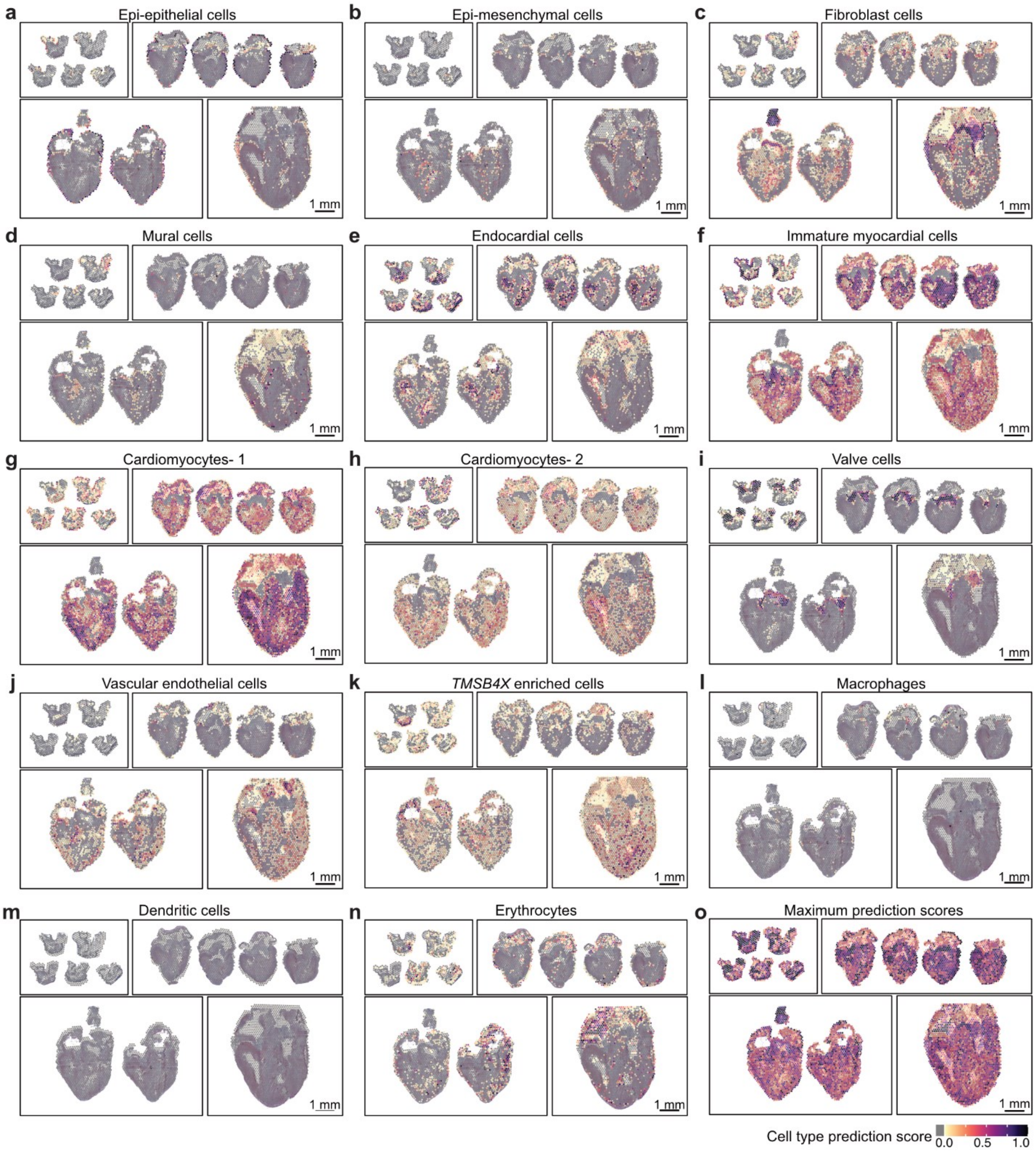

Supplementary Figure 2: Anchor based integration of scRNA-seq and spatial RNA-seq and label transfer
Spatial cell type prediction scores derived by transferring scRNA-seq cell types on spatial RNA-seq data using an anchor based integration and batch correction method (Seurat-v3). Only Visium spots located under tissue are shown. **a)** Epicardial progenitor cells-1 localized to the outer epicardial layer in day 7 and day 10 stages. **b)** Epicardial progenitor cells-2 localized to inner myocardium across stage but not outer epicardial layer. **c)** Fibroblast-like cells localized to valve regions and outer epicardial layer at day 14 and within myocardium in day 10 and day 14. **d)** Mural cells localized to inner myocardium at day 10 and 14 stage. **e)** Endocardial cells localized to ventricular endocardium across stage. **f)** Immature myocardial cells localized in early day 4 and 7 stage and decreases in day 10 and 14 stage. **g)** Cardiomyocytes-1 localized to all stages in myocardium but increases with later stages of day 10

and 14. **h)** Cardiomyocytes-2 localized to all stages in myocardium. **i)** Valve cells localized to valve regions across stage but lower in later day 14 stage. **j)** Vascular endothelial cells localized to myocardium in later day 10 and 14 stages. **k)** *TMSB4X* high cells localized to myocardium and increases with day 10 and 14 stages. **l)** Macrophages localized to sparse locations in myocardium across stage. **m)** Dendritic cells were not localized to any locations across stage suggesting they are circulating cells. **n)** Erythrocytes localized in atrial and ventricular chambers. **o)** Maximum cell type prediction scores across stage.

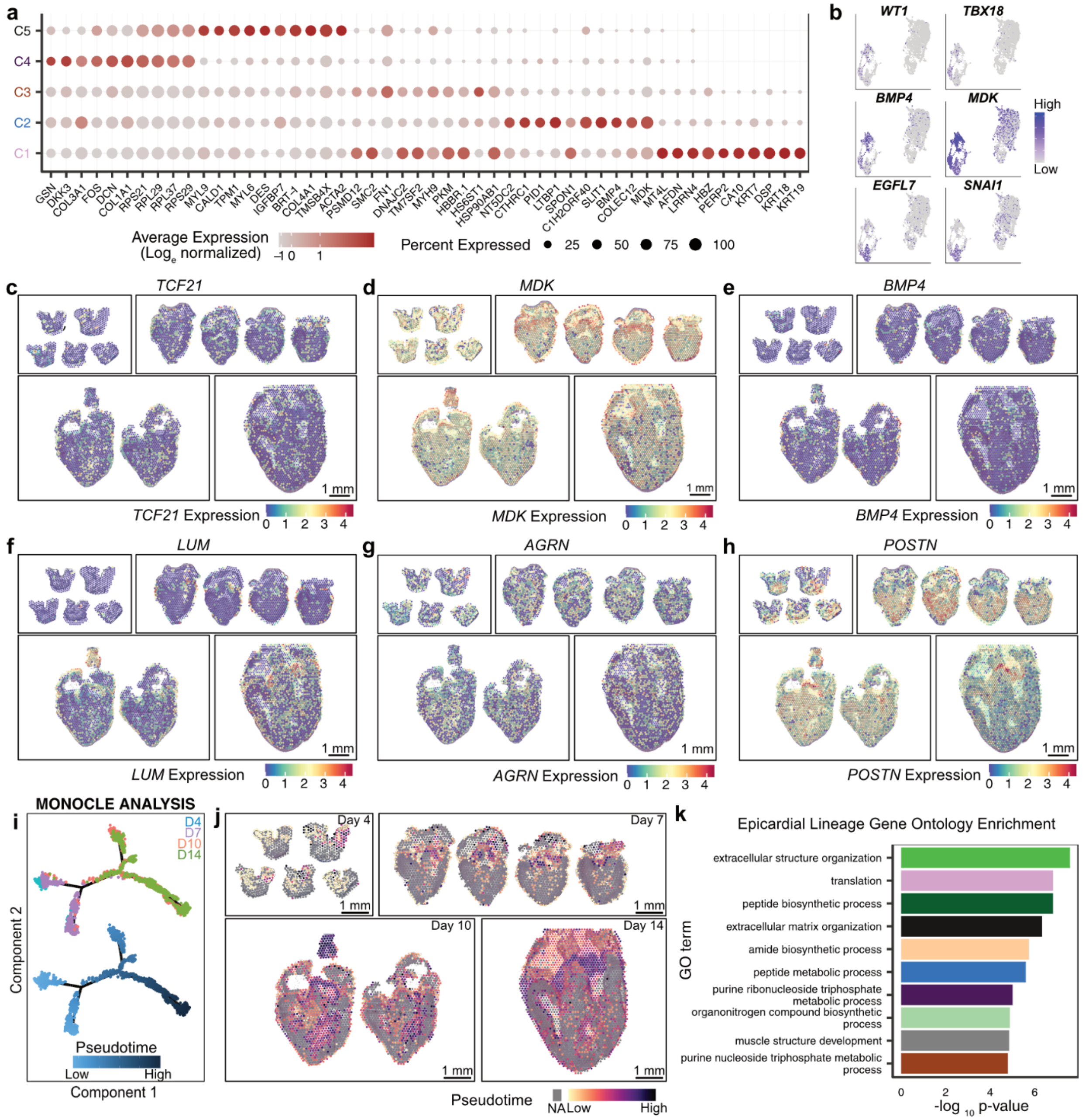

Supplementary Figure 3: Epicardial lineage analysis a) Dot plot of the top ten differentially expressed genes per epicardial subcluster. Size of the dot represents the percent of cells in the clusters expressing the marker and the color intensity represents the average expression of the marker in that cluster. **b)** Feature plots showing expression of more epicardial related markers. **c, d, e, f, g, h)** Spatial RNA sequencing gene expression plots for featured gene markers. **i)** Monocle v2 DDR-Tree projection of scRNA-seq cells from individual epicardial lineage labeled by developmental stage (top) and monocle pseudotime (bottom). **j)** Spatially resolved spatial RNA-seq spot pseudotime (monocle-based) for epicardial lineage across developmental stages. Spot pseudotime was estimated using a similarity map between scRNA-seq cells and spatial RNA-seq spots. **k)** Top 10 gene ontology (GO) terms for genes significantly correlated with pseudotime in epicardial lineage. Significant genes for gene ontology (GO) analysis were selected using differential marker analysis between sub-clusters within lineages with p-value threshold $< 10^{-15}$.

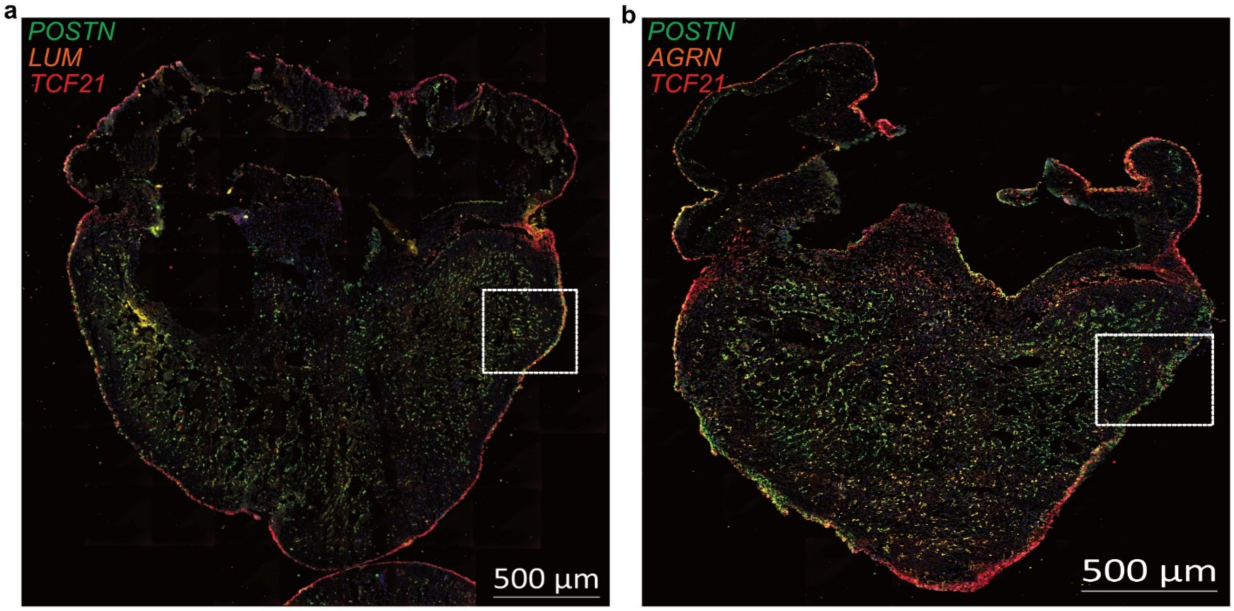

Supplementary Figure 4: Single molecular fluorescent in situ hybridization of epicardial lineage analysis a) smFISH images of day 7 (HH31) whole hearts for *POSTN*, *LUM*, *TCF21* in one section. **b)** smFISH images of day 7 (HH31) whole hearts for *POSTN*, *AGRN*, *TCF21* in one section. Blue channel is DAPI. Representative images of three to four biological replicates.

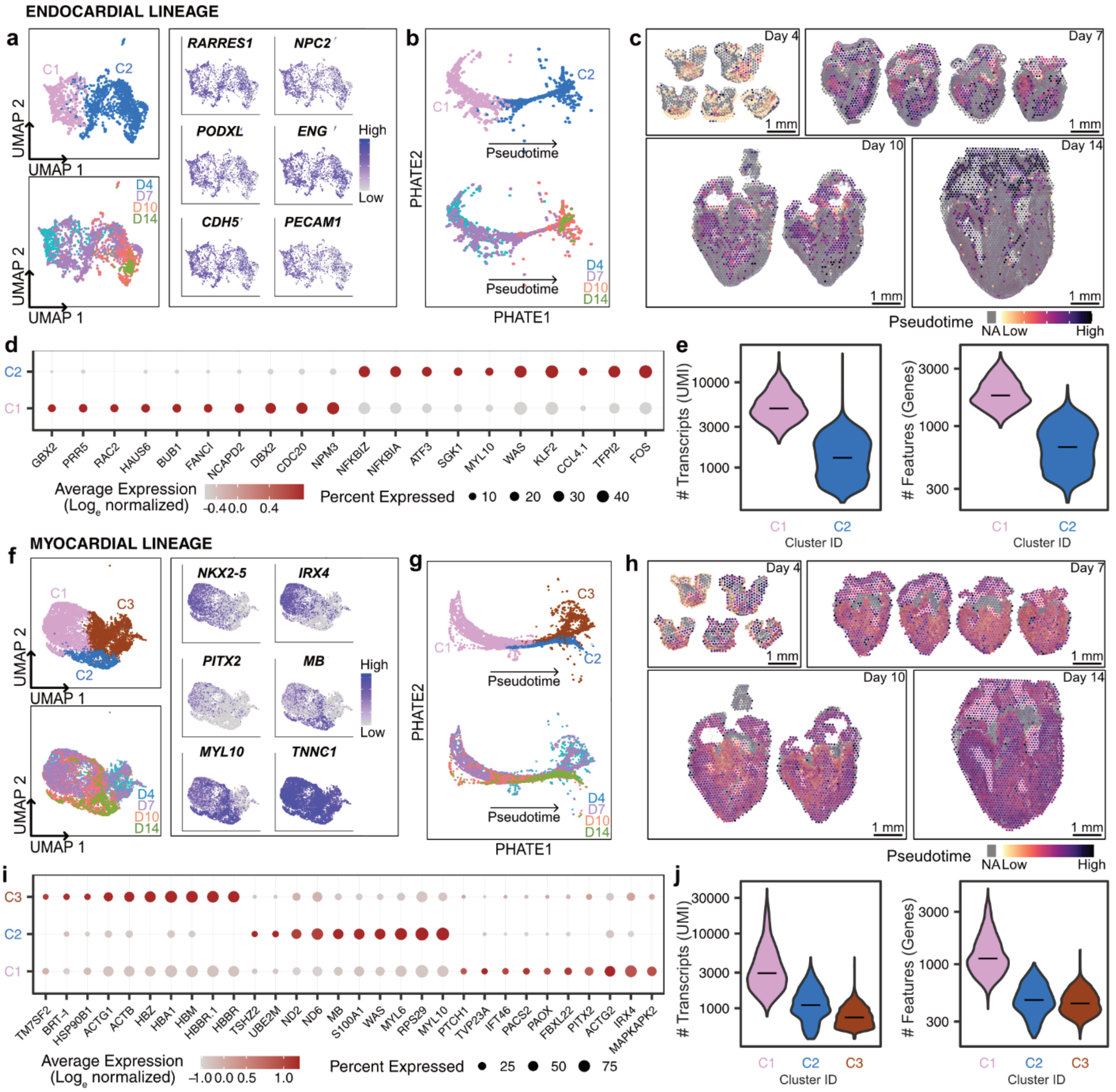

Supplementary Figure 5: Endocardial and myocardial lineage analysis. a) UMAP projection of single-cell transcriptomes from individual endocardial cells clustered by gene expression and colored by cell type (left-top) and developmental stage (left-bottom). Feature plots showing expression of gene markers for individual endocardial cells (right). **b)** Endocardial cells visualized by PHATE and labeled by cell type (top) and development stage (bottom). **c)** Spatially resolved spatial RNA-seq spot pseudotime for endocardial lineage across developmental stages. Spot pseudotime was estimated using a similarity map between scRNA-seq cells and spatial RNA-seq spots. **d)** Dot plot of top ten differentially expressed genes for endocardial subclusters. Size of the dot represents the percent of cells in the clusters expressing the marker and the color intensity represents the average expression of the marker in that cluster. **e)** Violin plot of the number of transcripts (left) and genes (right) expressed by each endocardial cell cluster. **f)** UMAP projection of single-cell transcriptomes from individual myocardial cells clustered by gene expression and colored by cell type (left-top) and developmental stage (left-bottom). Feature plots showing expression of gene markers for individual myocardial cells (right). **g)** Myocardial cells visualized by PHATE and labeled by cell type (top) and development stage (bottom). **h)** Spatially resolved spatial RNA-seq spot pseudotime for myocardial lineage across developmental stages. Spot pseudotime was estimated using a similarity map between scRNA-seq cells and spatial

RNA-seq spots. **i)** Dot plot of top ten differentially expressed genes for myocardial subclusters. Size of the dot represents the percent of cells in the clusters expressing the marker and the color intensity represents the average expression of the marker in that cluster. **j)** Violin plot of the number of transcripts (left) and genes (right) expressed by each myocardial cell cluster.

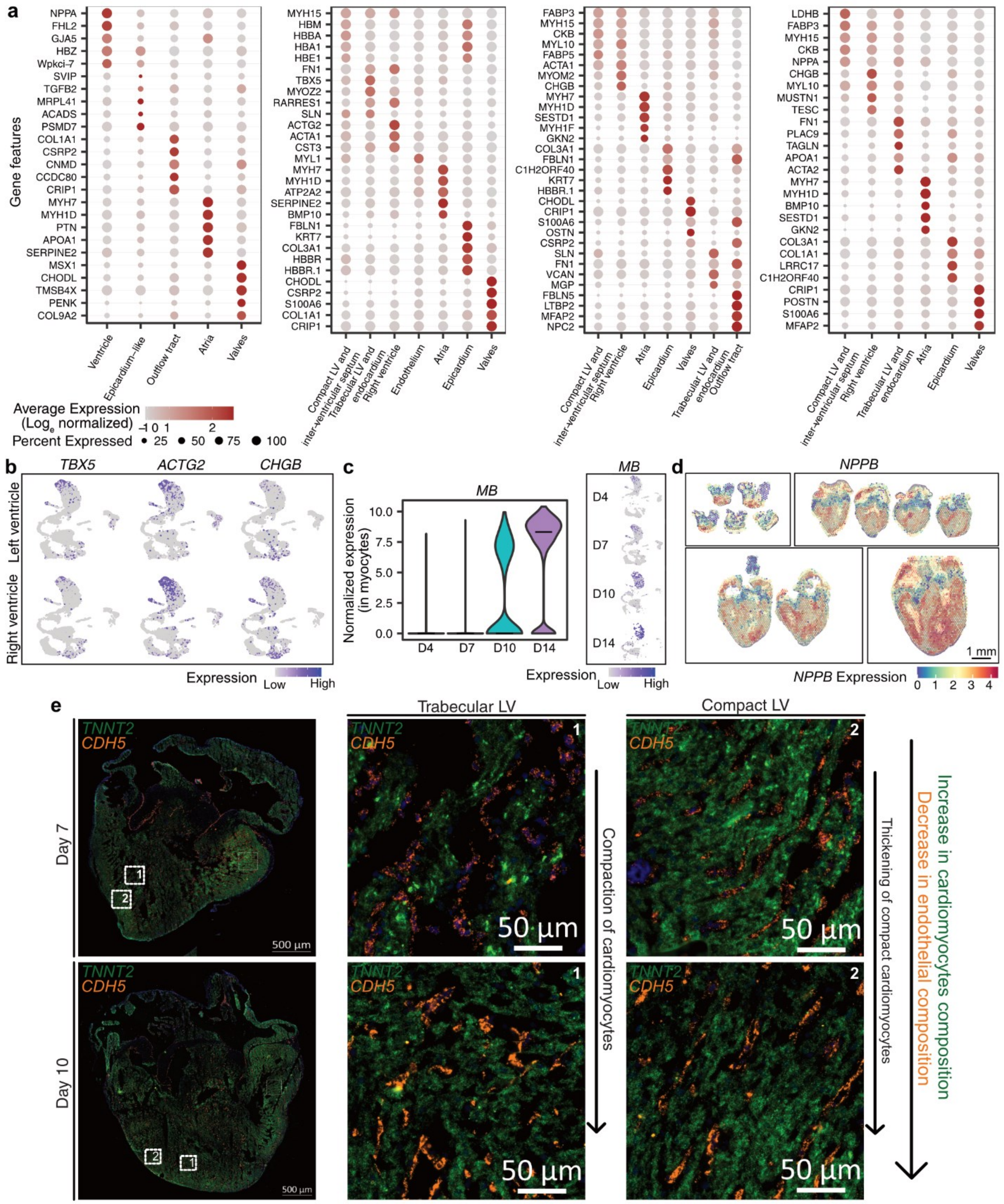

Supplementary Figure 6: Spatial RNA-seq reveals spatially restricted genes in cardiac tissue during development. a) Gene expression of anatomical region specific differentially expressed genes in spatial RNA-seq data across developmental stages. Regions were labelled by clustering of gene expression for barcoded spots in spatial RNA-seq data. Size of the dot represents the percent of spots in the spot clusters expressing the gene marker and the color intensity represents the average expression of the gene markers in that spot cluster. **b)** Feature plots showing the normalized scRNA-seq expression of *TBX5*, *TBX20*, and *ACTG2* genes in cells from left and right cardiac ventricles.

c) Violin plot showing the distribution of normalized scRNA-seq expression of myoglobin (*MB*) in myocardial lineage across developmental stages. Insets show normalized *MB* expression across stages in the entire scRNA-seq dataset. **d)** Spatially resolved normalized gene expression for *NPPA* across all four developmental stages. **e)** smFISH stained day 7 and day 10 hearts for cardiomyocyte marker (*TNNT2*) and endothelial marker (*CDH5*) demonstrating differences in trabecular versus compact myocardium regions. Representative images of three to four biological replicates.

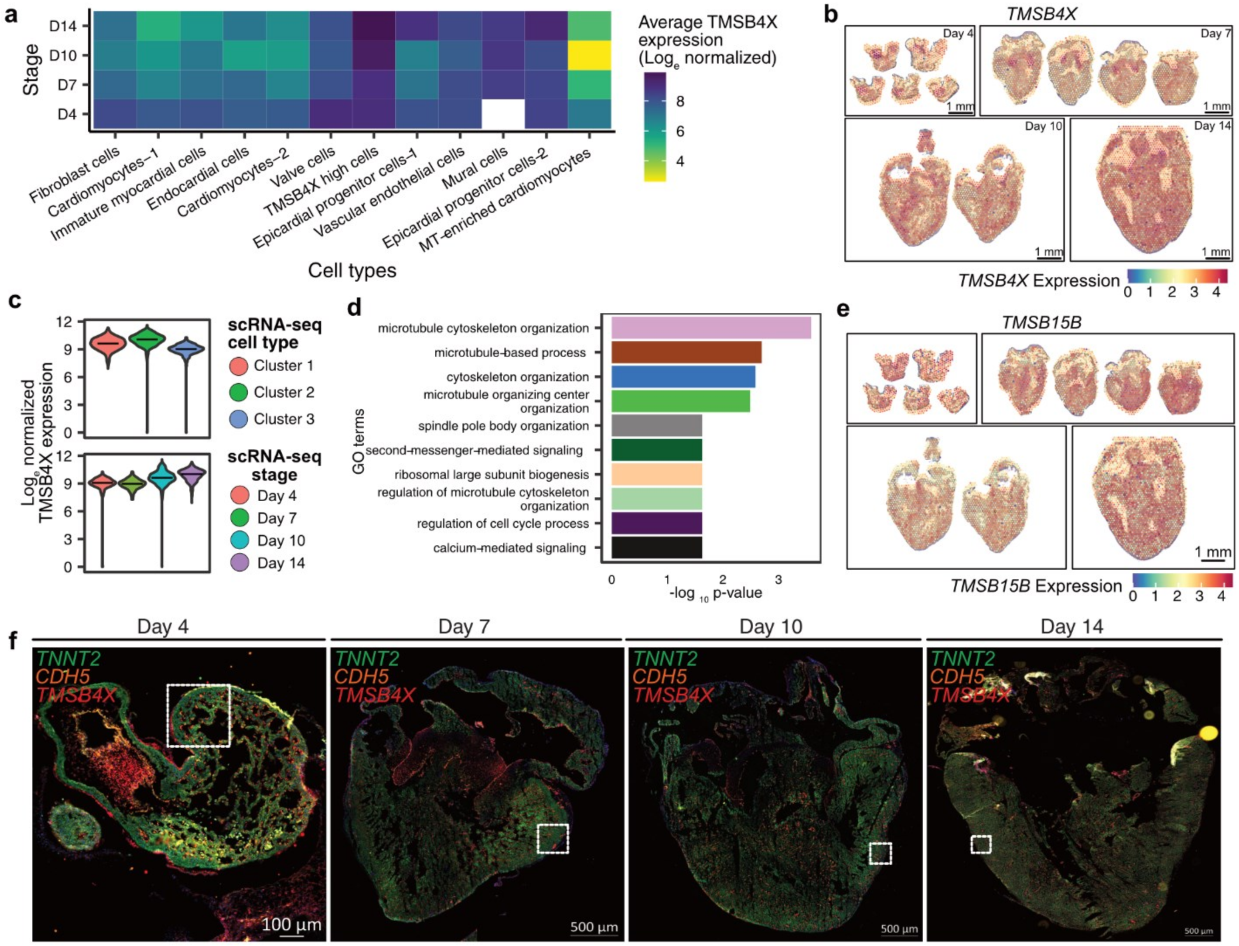

Supplementary Figure 7: Characterization and validation of cell types in TMSB4X high cluster. **a)** Log-normalized gene expression of thymosin beta-4 (*TMSB4X*) in scRNA-seq cell type clusters across four developmental stages. **b)** Spatially resolved thymosin beta-4 (*TMSB4X*) normalized gene expression across stages in chick heart spatial RNA-seq data. **c)** Log-normalized gene expression of thymosin beta-4 (*TMSB4X*) in subclusters within the "*TMSB4X* high cells" scRNA-seq cell cluster. *TMSB4X* expression is labeled by subcluster (top) and developmental stage (bottom). **d)** Top 10 gene ontology (GO) terms for genes significantly enriched in *TMSB4X* high cells scRNA-seq cluster. Significant genes for gene ontology (GO) analysis were selected with p-value threshold $< 10^{-15}$. **e)** Spatially resolved thymosin beta 15B (*TMSB15B*) normalized gene expression across stages in chicken heart spatial RNA-seq data. **f)** smFISH whole heart sections across all four developmental stages for *TNNT2* (green), *CDH5* (orange), *TMSB4X* (red), and DAPI (blue). Representative images of three to four biological replicates.

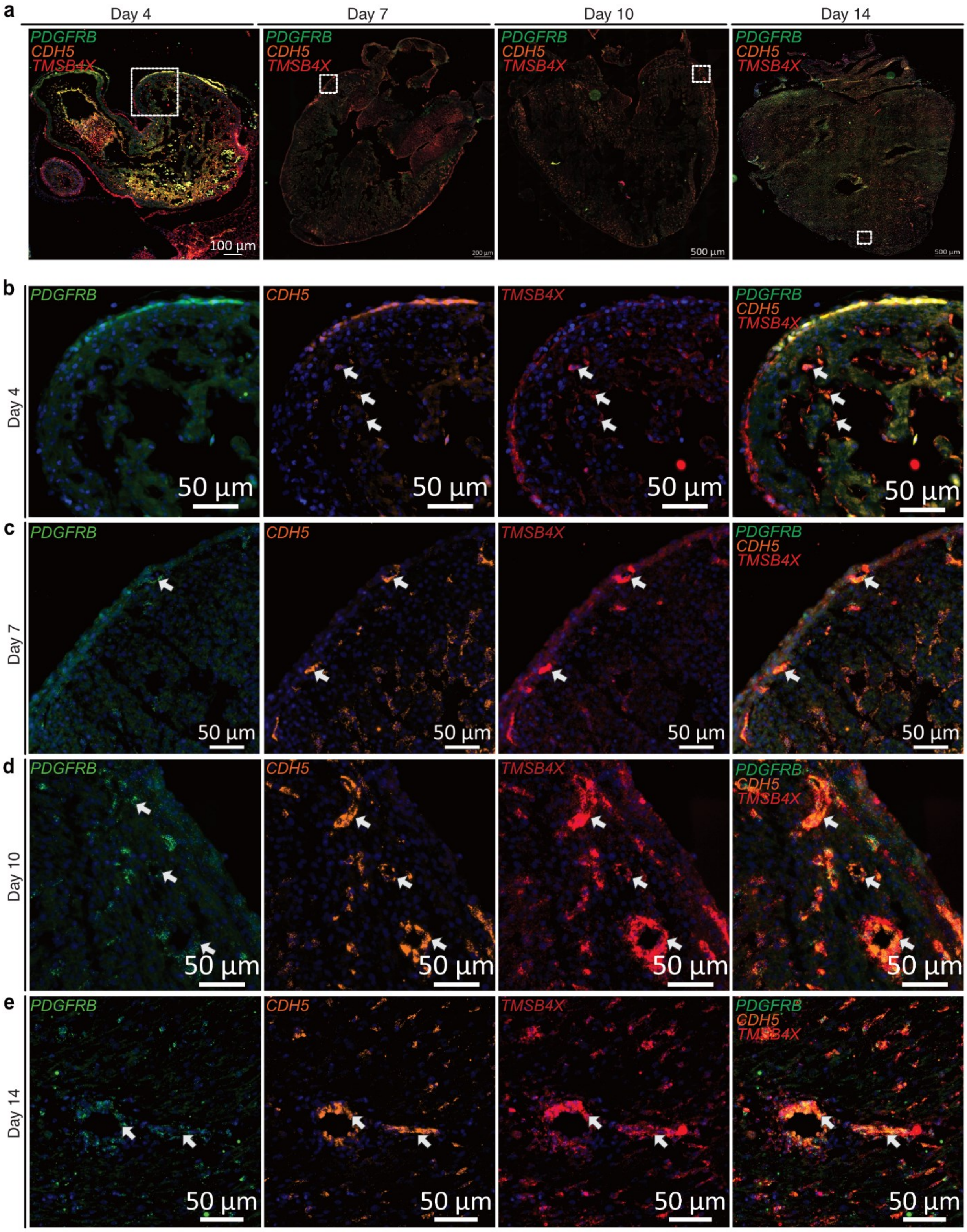

Supplementary Figure 8: Single molecular in situ hybridization demonstrating prevalent TMSB4X expression in coronary vascular cells **a)** smFISH images of chicken heart ventricular free wall sections across four developmental stages labeled for mural cell marker *PDGFRB* (green), endothelial cell marker *CDH5* (red), thymosin

beta-4 *TMSB4X* (red), and DAPI (blue). Representative images of three to four biological replicates. **b)** day 4 (HH24); Cells with high autofluorescence in all channels are Erythrocytes, **c)** day 7 (HH31), **d)** day 10 (HH36), **e)** day 14 (HH40).

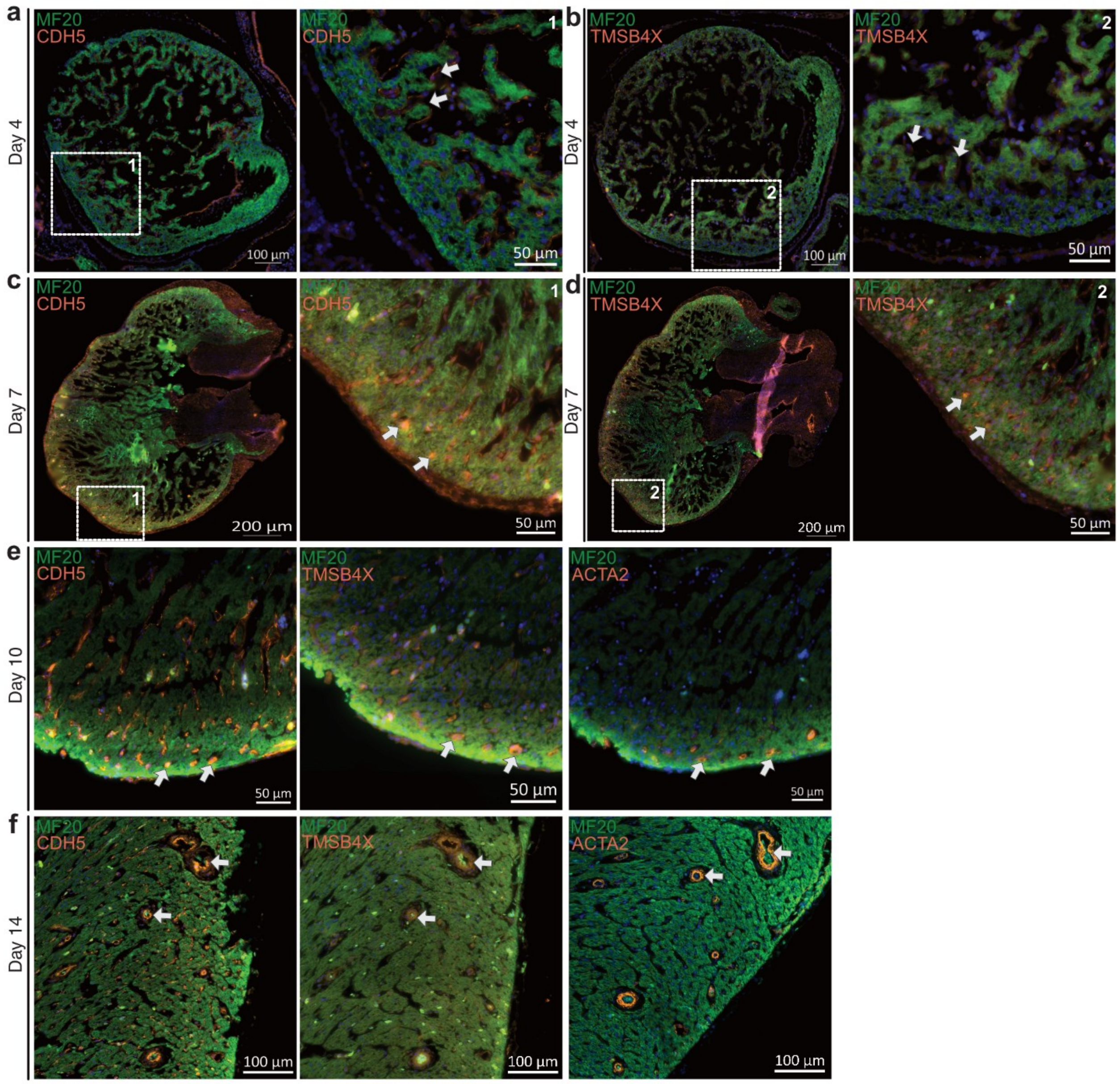

Supplementary Figure 9: Immunohistochemistry staining to characterize thymosin beta-4 protein expression across all four developmental stages. a) Immunohistochemistry (IHC) staining of day 4 whole hearts (right) and ventricular free wall (left) for cardiomyocyte cell marker MF20 (green), endothelial marker CDH5 (orange), and DAPI (blue). **b)** IHC staining of day 4 whole hearts (left) and ventricular free wall (right) for MF20 (green), thymosin beta-4 TMSB4X (orange), and DAPI (blue) on the sister section. **c)** IHC staining of day 7 whole heart (left) and ventricular free wall (right) for MF20, (green), CDH5 (orange), and DAPI (blue). **d)** IHC staining of day 7 whole heart (left) and ventricular free wall (right) for MF20, (green), TMSB4X (orange), and DAPI (blue) on sister section. **e)** IHC staining of day 10 ventricular free wall on sister sections for MF20, (green), CDH5 (orange), and DAPI (blue) (left), IHC staining of day 10 ventricular free wall on sister sections for MF20, (green), TMSB4X (orange), and DAPI (blue) on sister section (middle), and IHC staining of day 10 ventricular free wall on sister sections for MF20, (green), smooth muscle marker ACTA2 (orange), and DAPI (blue) on sister section (right). **f)** IHC staining of day 14 ventricular free wall on sister sections for MF20, (green), CDH5 (orange), and DAPI (blue) (left), IHC staining of day 14 ventricular free wall on sister sections for MF20, (green), TMSB4X (orange), and DAPI (blue) on sister section (middle), and

IHC staining of day 14 ventricular free wall on sister sections for MF20, (green), smooth muscle marker ACTA2 (orange), and DAPI (blue) on sister section. All representative images of three to four biological replicates (right).

Supplementary Note 1: Differences between trajectory reconstruction using monocle v-2 and PHATE

Monocle 2 uses a technique called "reversed graph embedding" to learn the structure of the manifold that describes a single-cell experiment data. Monocle performs a dimensional reduction of gene expression data using a discriminative dimension reduction approach, while preserving the local structure in data. While reconstructing lineages, monocle gives the user an option to choose a set of genes that define the biological process, which has a major impact on the shape of the trajectory. PHATE on the other hand is a visualization method that captures both local and global nonlinear structure in the data using an information-geometric distance between data points. PHATE denoises the data while performing dimensional reduction, and therefore can be performed directly on the whole transcriptome dataset.
