# Supplementary material for: Spatiotemporal single-cell RNA sequencing of developing chicken hearts identifies interplay between cellular differentiation and morphogenesis
Source: Nat Commun. 2021 Mar 19;12:1771. doi: 10.1038/s41467-021-21892-z (PMC7979764; doi:10.1038/s41467-021-21892-z)
Supplement: Supplementary file 6 — Description of Additional Supplementary Files [file 41467_2021_21892_MOESM6_ESM.pdf]

Title: Supplementary Data 1

Description: Canonical gene markers used for labelling scRNAseq cell type clusters (Section 1).

Title: Supplementary Data 2

Description: Additional gene markers used for labelling lineage subclusters during trajectory reconstruction analysis (Section 2).

Title: Supplementary Data 3

Description: Target split probe sequences and initiator split sequences for imaging gene transcripts using Hybridization chain reaction (HCR) v3: single molecule RNA FISH approach.

Title: Supplementary Data 4

Description: Initiator sequences and fluorophore combinations used for imaging gene transcripts using Hybridization chain reaction (HCR) v3: single molecule RNA FISH approach.
